# Unmasking the Enigma of Cerebral Palsy: A Traditional Review

**DOI:** 10.7759/cureus.11004

**Published:** 2020-10-17

**Authors:** Bryan A Ikeudenta, Ian H Rutkofsky

**Affiliations:** 1 Psychiatry, Neuroscience, California Institute of Behavioral Neurosciences & Psychology, Fairfield, USA

**Keywords:** cerebral palsy, spasticity, treatment of cerebral palsy, cerebral palsy and pathophysiology, birth asphyxia, intellectual disability, cerebral palsy types, cerebral palsy and anxiety

## Abstract

Cerebral palsy is a group of neuromuscular diseases that is primarily common in the pediatric population and is the most common cause of neurological and motor disability in children. Cerebral palsy is comprised of various subtypes with the most common type being spastic cerebral palsy. It is highly associated with prematurity and affects nerve function, motor function, and intellectual capacity. It is also associated with nutritional deficiencies and gastrointestinal dysfunction. Cerebral palsy is diagnosed via clinical evaluation and does not have specific laboratory or image findings, but certain imaging findings are positively correlated with it. There are numerous interventions and treatment modalities that are aimed at ensuring the highest quality of life for the patient and their families.

This article was compiled with peer-reviewed publications from the PubMed database in which various keywords were utilized in the search engine. These peer-reviewed articles were selected without geographical restrictions and selected based on the use of the English language. These articles were also selected on the restriction of publication within the last 10 years.

This review article on cerebral palsy will serve as a medium of education for the physician, healthcare team, and family involved in the management of children or adults with cerebral palsy. It is important because it discusses the possible etiologies, diagnostic and assessment techniques, prevention methods, and possible rehabilitation interventions. This article aims to broaden the reader's understanding of cerebral palsy and answer any questions that may arise during the management of this disease. The management of cerebral palsy is often plagued with frustration, depression, and anxiety. The main goal of treatment is to attain the highest quality of life for the family and the child.

## Introduction and background

The term cerebral palsy refers to a group of neurodevelopmental disorders that are known for causing dystonia, motor, and movement dysfunction, and intellectual disability [1]. This disorder commonly occurs in two to three out of 1,000 live births and has multiple etiologies resulting in the corresponding brain injuries [1,2]. A myriad of movement disorders is seen with cerebral palsy and include spasticity, dyskinesia, ataxia, or mixed types involving the mentioned types. Spasticity or spastic cerebral palsy is the commonest form of this movement disorder and occurs in 80% of children diagnosed with cerebral palsy [3]. Cerebral palsy can also be associated with different co-morbidities which include; behavioral disturbance, cognitive difficulties, communication difficulties, sensory impairments, epilepsy, and intellectual disability. Cerebral palsy is almost always diagnosed in early childhood, but its course can continue into adolescence, adulthood, and have a varying spectrum of severity. Evidence supports that children diagnosed with cerebral palsy have a higher rate of deterioration in physical and mental functioning and are at an increased risk for secondary disease [4].

Intellectual disability affects more than a third of individuals diagnosed with concomitant cerebral palsy [4]. The degree of intellectual disability also owes to the underlying difficulties in diagnosing mental illnesses in cerebral palsy. In other words, the challenging masking of symptoms of cerebral palsy overshadows the correct diagnosis of mental illness. This could mean the incidence of mental illnesses will be higher for those with cerebral palsy because of their lack of typical expressiveness [4]. Some clinical research studies have taken into consideration various preventative measures that may reduce the acquisition of cerebral palsy. There are also diagnostic and treatment modalities that have been illustrated to better have an understanding of this chronic condition and its preventability [5]. These efforts have improved the efficiency of observational and experimental epidemiologic studies related to prevention, as well as intervention trials for individuals with cerebral palsy [6].

This article aims to review clinical research on the causes, diagnosis, treatment of cerebral palsy, and create awareness about this disease. We will begin by reviewing research on the types of cerebral palsy, its diagnostic criteria, and its severity concerning motor dysfunction, life quality, imaging studies, and treatment. This discussion will be of keen interest to physicians who are in charge of the care of cerebral palsy patients and the parents of children that have been diagnosed with cerebral palsy.

## Review

Method

The composition of this review article consisted mainly of the PubMed database where this engine was used to search for publications using regular keywords that illustrated studies tackling cerebral palsy pathophysiology, symptoms, diagnosis, treatment, possible preventive measures, and impact on life. The search yielded numerous articles including editorials, review articles, free full texts, and abstracts. After thoroughly reviewing these, relevant publications and their references were utilized to search for other publications. 

These review articles were chosen based on the following inclusion criteria: publication in English language, articles published within the last 10 years, human subjects and observational studies including review articles. These publications were also chosen without geographical or international restrictions. Conversely, articles were exempted based upon the fact being written in languages other than English, greater than 10 years since publication, non-human studies, and study type being meta-analyses or case series. 

The keywords used to search included the following; cerebral palsy, spasticity, treatment of cerebral palsy, cerebral palsy and pathophysiology, birth asphyxia, intellectual disability, and cerebral palsy, cerebral palsy types, cerebral palsy, and anxiety. The use of these keywords yielded: 12,556 peer-reviewed published articles for cerebral palsy, 10,730 peer-reviewed articles for spasticity, 7,498 peer-reviewed articles for treatment of cerebral palsy grouped, 6,823 peer-reviewed articles for keywords diagnosis of cerebral palsy grouped, 3,598 peer-reviewed articles for keywords cerebral palsy and pathophysiology grouped, 2,519 peer-reviewed articles for birth asphyxia, 580 peer-reviewed articles for keywords intellectual disability and cerebral palsy grouped, 432 peer-reviewed articles for keywords cerebral palsy types grouped, and 110 articles for keywords cerebral palsy and anxiety grouped.

After applying the inclusion and exclusion criteria were and in unison with the keywords used on the PubMed search database, a total of 39 publications including abstracts and review articles were used for this paper. 

Discussion

The Role of Birth Asphyxia and Perinatal Infections in Cerebral Palsy

Cerebral palsy coins a group of neuromuscular disorders that affect movement, posture, cognition, and quality of life. It the most common cause of disability among the pediatric population. The understanding of the causes, pathophysiology, and complications of cerebral palsy will greater aid the formation and utilization of efficient preventative modalities.

The most common etiology of cerebral palsy is birth asphyxia which is referred to as ischemia or an infarction resulting from a decrease in oxygenation to the brain and subsequent neurological damage [7-9]. This risk of this insult is increased during any point in pregnancy or post delivery but is highest at birth [9]. There are different scenarios where birth asphyxia can be concluded as the potential cause of cerebral palsy. A pregnancy could be complicated by a placental abruption that results in the delivery of a fetus that is pale, apneic, and unresponsive to external stimulation [9]. One can conclude that an asphyxial event has occurred due to the interruption in oxygen supply to the brain. Another event can occur with the same presentation but no direct cause of ischemia to the brain e.g., perinatal infection. In this event, intrauterine or perinatal infection or inflammation can be synonymous in clinical presentation to birth asphyxia [10]. Infants who are exposed to these antecedents can exhibit varying levels of consciousness during the first breaths of life and are at an increased risk for seizures and ensuing neurological disabilities [9,10]. This risk is further increased in the event of placental abruption or fetal-maternal insufficiency and superimposed intrauterine growth restriction [11]. Research also suggests that the aforementioned events are also a potential cause of neonatal encephalopathy and spastic cerebral palsy that may mimic birth asphyxia [12,13].

Cerebral palsy is a group of debilitating neuromuscular diseases that most commonly occur during pediatric life and are associated with different antenatal, intrapartum, and neonatal risk factors [14]. In the realm of cerebral palsy, some models illustrate a pathway of risk factors that are centered around placental insufficiency and maternal infection. These may lead to unique presentations that are closely related to birth asphyxia. Understanding these factors can greater reduce the incidence and complications of this group of neuromuscular diseases.

Classifications of Cerebral Palsy and Their Effects on the Quality of Life

Cerebral palsy is a group of neuromuscular diseases commonly complicated by neurocognitive disability, with spastic type being the most frequent type identified and the second most common type being the dyskinetic type, which is known for its highly disabling features [15]. In recent times, a classification system for cerebral palsy subtypes did not exist. It was common practice to group or describe cerebral palsy solely upon assessment of the motor disorder and regions of the body affected, for example, spastic quadriplegia or hemiplegia. The description or grouping of cerebral palsy needs to incorporate different factors like severity, associated disorders, and subsequent impairments [15]. Describing the motor disorder in cerebral palsy was made in terms of muscle tone (can hypotonia or hypertonia) or on movement or muscular disturbance (bradykinesia, dyskinesia, spasticity, or ataxia) or in unison with both [15,16]. Different cerebral palsy subtypes can have predominately mixed presentations or pure presentations [16].

The subtypes of cerebral palsy include spastic, dyskinetic, ataxic, and mixed forms of cerebral palsy. Spastic cerebral palsy causes hypertonia, hyperreflexia in the form of muscular spasms and often painful muscles. Spastic type can be further broken down into spastic diplegia which classically involves hypertonia of the leg muscles; hemiplegia, which affects half of or a single side of the body with movement disturbances seen mostly in the upper extremity and quadriplegia which affects both arms and both legs [16]. Spastic quadriplegia is the most severe form of spastic cerebral palsy and is often complicated by spinal deformities, language, feeding disorders, seizures, and muscle contractures.

The second most common type of cerebral palsy is the dyskinetic type and is associated with abnormal, uncoordinated movements of both upper and lower extremities. This type of cerebral palsy is often associated with variations in muscular tone e.g. dystonia, athetosis, or chorea [17]. These movement disorders can exist solely or in combination and are complicated predominately by issues with fine motor skills.

The ataxic type of cerebral palsy is another form of cerebral palsy with uncoordinated, jerky movements [17]. This type of cerebral palsy is often associated with speech impairment, ocular movement dysfunction, and difficulty swallowing. The classic system of grouping cerebral palsy provided little to no guidance into the limitations of the child, severity of the disease, or on quality of life. It ideally served as a benchmark in describing physical symptoms. Keen interests have been formed in looking into the function, activity limitations, severity, and quality of life. This has led to the formation of function-based classification groups. An example is the Gross Motor Function Classification (GMFCS) [18]. This classification group incorporates motor function and aims to address differences between the subtypes of cerebral palsy as it relates to individual capabilities e.g. ambulation. The GMFCS is divided into five classes or levels: Level I - ambulation without restrictions, Level II - ambulation without aid de­vices, but with some limitations outside the house, Level III - ambulation with assistance, Level IV - ambulation via assistance with powered devices or trolley, Level V - little to no ambulation [18,19].

The degree of independent ambulation is the most common variable used when assessing the quality of life or independent function capabilities of a cerebral palsy patient. This is also the most common question asked by parents at the time of diagnosis [19]. Recent studies into functionality have shown that those diagnosed with spastic diplegia or hemiplegia have a higher incidence of independent ambulation when compared to those diagnosed with spastic quadriplegia [20]. Therefore, it can be said that those patients who can independently ambulate have a higher chance of living a higher quality of life when compared to those that ambulate less or are unable to ambulate.

Cerebral palsy can be complicated by nutritional imbalances and patients can have variations in weight class [21]. This disparity is commonly assessed through the use of pediatric growth charts. Primary care providers often treat more patients who fall in the underweight category [21]. When the patient is not meeting a specific percentile in weight and height over a certain period, the child could be diagnosed with failure to thrive. This diagnosis can potentially be inaccurate if other factors like disease activity or clinical progression are not considered. Malnutrition has several impacts on disabled individuals but misdiagnosis of failure to thrive is consequential [21,22].

Patients with cerebral palsy are seen to grow at a slower rate when compared to typical developmental milestones even in the presence of optimum healthcare and nutrition [22]. This could be perceived by the caretaker as them not doing enough and initiating unwarranted adjuvant feeding therapy. Rapid weight gain schemes can lead to metabolic syndrome and other clinical sequelae especially in cerebral palsy patients where difficulty swallowing, encopresis, gastroesophageal reflux, and bouts of emesis are common [21-24].

Cerebral palsy has different subtypes and is normally classified on motor symptoms and the topographical area of these symptoms. This classic way of grouping these disorders relayed little insight into functional status and quality of life. A newer classification method known as the GMFCS sheds light on this group of diseases for functionality among the numerous subtypes. Functionality is usually measured as a metric of the degree of independent ambulation. It was concluded that for spastic cerebral palsy, some degree of independent ambulation was achieved among the diplegic and hemiplegic types when compared to spastic quadriplegia [20]. Cerebral palsy is also associated with variations in weight and height when compared to the general population. Cerebral palsy patients are seen to be more underweight and care needs to be taken to ensure that the patient is looked at in their totality to ensure that consequences of over nutrition are not experienced.

Diagnosis of Cerebral Palsy

Diagnosing cerebral palsy is essentially clinical without any specific laboratory or imaging studies. Although, certain imaging findings can be highly correlated with cerebral palsy [25]. A diagnosis of cerebral palsy is also supported by multiple observatory findings by the parent and attainment of specific milestones like pulling to stand, posture, and walking [8]. The diagnosis of cerebral palsy is also related to their degree of prematurity at birth. Neurological abnormalities in premature children may not be associated with motor dysfunction are often transient. A definitive diagnosis is only made after repeated examinations and not a single instance of abnormal exam findings [26,27].

Imaging studies of infants play an ancillary role for diagnosis. In preterm infants, the main imaging study of choice was the neonatal ultrasound. In recent years, the diagnostic imaging test of choice has shifted to magnetic resonance ultrasound (MRI) which is highly specific for intracranial findings. The most common associated MRI findings were periventricular leukomalacia which is highly associated with prematurity and focal infarction [27].

The sensitivity of diagnosing cerebral palsy is improved when different tools are brought into play. These diagnostic tools include neuroimaging, neurological and neuromotor exams [28]. In addition to the above, I will shed some light on the neurological assessments and their influence on the diagnosis. Neurological assessments are routinely used to monitor and screen for abnormalities in the growing infant. The best known are the Dubowitz, Hammersmith, Prechtl, Touwen, and Amiel-Tison neurological assessments [28]. The main assessment is the Hammersmith assessment and its ideal benefit was that it identified those children at the highest risk of cerebral palsy and provided information on the type of disease and its subsequent severity [28]. The assessment also addressed overall neurological function i.e. muscle tone, reflexes, cranial nerves, and behavior [29]. This is a useful tool that allows the physician to follow these infants over their developing years post birth and identify alarming symptoms or normal development.

A tool known as the general movement assessment (GMA) is the most efficacious method to assess neuromotor function [29]. This tool looks at the general movements of the post-term infant and provides information about the brain and its connection to the periventricular white matter [30]. General movements are assessed based on their frequency, variation, and complexity with abnormal movements being noted as reduced or even absent [29-31].

Cerebral palsy is most commonly diagnosed during pediatric life and is based upon multiple clinical encounters and symptom presentation. It is a clinical diagnosis not dependent on any lab studies or imaging but does have strongly associated imaging findings. Diagnosing cerebral palsy is ideally done in conjunction with neurological and motor assessments that are suited to establish the relationship between the risk of the disease and its severity. This is important because the identification of this disease early can guide management of this disorder and affect quality of life.

Prevention and Treatment of Cerebral Palsy

Cerebral palsy is highly associated with prematurity and suboptimal birth weight [5]. This could be due to maternal comorbidities, intrauterine or extrauterine insults [6,7]. The mainstay of reducing the incidence of this disease should be aimed at addressing those causes and their risk factors. There are a vast number of these factors and they all share a common thing; they are potentially preventable [8]. These risk factors include maternal age (low or advanced), nulliparity or multiparity, maternal disease like diabetes, hypertension, preeclampsia, eclampsia, placental abruption, and intrauterine infections [14]. There are also risk factors that are associated post-delivery and they are acute hypoxic events stemming from cardiovascular or respiratory diseases, strokes, seizures, prolonged ventilation, sepsis, metabolic or endocrine disorders and inborn errors of metabolism [15]. These risk factors highlight an umbrella of causal factors mainly being maternal-placental-fetal insufficiency and infection. Understanding these pathways are crucial in the prevention and treatment of cerebral palsy.

Interventions for cerebral palsy are aimed at enhancing development, improving parental awareness, education, and psychosocial support [32]. This helps to reduce anxiety and depression concerning the child's diagnosis. The psychosocial aspect of the intervention is aimed at the provision of goals or milestones for intervention [32]. This allows for dialogue on fears, frustrations, and individualized goal metrics for self-care and sufficiency i.e. feeding and bathing and reduced parental stress [33]. This owes to the fact that a family incorporated form of therapy improves the quality of life of the child with cerebral palsy [32,33]. This concludes that family and parental psychosocial support are effective interventional measures.

Parental guidance about adequate chronic illness control, adequate antenatal and postdelivery care, prevention of intrauterine infection are efficient measures to reduce the incidence of premature birth, low tier birth weight, and subsequent neurologic abnormalities. During pregnancy, it has been studied that magnesium sulfate given at 32 weeks' gestation is neuroprotective and greatly reduces the risk of neurological insults like cerebral palsy [33]. Caring for a child with cerebral comprises of lifetime diverse specialist management that aims to give the family and child the greatest quality of life. It is a process that involves rehabilitation and psychosocial support. The rehabilitation aspect of care is dependent upon how early it is implemented and is guided by neuroplasticity. Neuroplasticity illustrates the central nervous system and its power to adapt upon exposure to different stimuli. The degree of change depends on the rehabilitation and these occur new responses after the rehabilitation scheme has been implemented. This owes to the fact these schemes have the greatest effect when the degree of plasticity is greatest i.e. during early life [34].

There are different forms of rehabilitation and they include motor, occupational, and neurological rehab [1,2]. Motor rehabilitation involves kinesiology which helps to restore lost motor function and gait through enforced motor activity. It also involves physiotherapy which helps to increase muscle strength, endurance, laxity of joints, and prevention or reduction of contractures [34]. These are facilitated by high and low-intensity exercises of the various muscle groups along with repetitive passive and action movements of the joints [34,35]. Occupational rehabilitation is aimed at improving critical thinking, problem solving, self-sufficiency, and cognition as these are relatively diminished in cerebral palsy [35].

Spasticity and hypertonia are the most common symptoms of cerebral palsy. These symptoms cause reduced joint laxity and add to the risk of contracture development [35]. It also reduces the goals of rehabilitation and overall quality of life. The treatment of spasticity involves additional physiotherapy and/or pharmacotherapy. The drug of choice for the treatment of spasticity is an antispasmodic known as baclofen [35]. Botox or botulinum toxin A is also another option but is reserved for cases of focal spasticity [36]. Refractory cases of spasticity may require surgical intervention. Some of these measures include intrathecal baclofen infusion via a pump, selective rhizotomy, or neurotomies [37]. Cerebral palsy is often complicated by nutritional disparities and gastroesophageal reflux and the appropriate adjuvant therapy should be started when applicable. Another common association with cerebral palsy is epilepsy [38]. Epilepsy associated with cerebral palsy is treated with medication as the gold standard of management. This form of epilepsy is often drug-resistant more commonly seen in spastic quadriplegia [39].

Cerebral palsy has a medley of risk factor associations in which a fair majority are deemed preventable. The treatment of this disorder is routed around rehabilitation which aims to bolster neurologic function, increase muscular dexterity, and improve overall cognition. There are also medical therapies that are indicated for the associated complications of cerebral palsy like spasticity and gastroesophageal reflux disease (GERD). This disease requires lifelong management and the main goal of treatment is the provision of the highest quality of life for the family and the child respectively.

Limitations

Cerebral palsy primarily affects muscle tone, coordination, and function with different effects on the central nervous system. A limitation that we had while compiling this review was researching its influence on intellectual capabilities. There were limited results on the effect of cerebral palsy on intellectual prowess. Another limitation that was encountered for this review was finding literature on the specific types of cerebral palsy. The results of our search of scientific literature on cerebral palsy being a group of disorders shed light on mostly the most common type in its group and deemphasized the other subtypes . Reducing these limitations would involve more investigations into the relationship between cerebral palsy and intellectual disability along with its various types.

## Conclusions

Cerebral palsy is a group of neuromuscular disorders and is the most common cause of disability within the first few years of life. It is most commonly due to birth asphyxia and with the highest risk due to prematurity. Cerebral palsy consists of different types with the most common type being spastic hemiplegia. This group of disorders is ideally diagnosed by clinical evaluation in the setting of multiple symptom occurrences. It is devoid of specific laboratory or imaging findings but is associated with certain positive image findings. Clinicians have come up with adjuvant methods of motor and neurological assessments that have been employed in describing the features of this disorder and its burden on life quality. Cerebral palsy has numerous interventions and management techniques with the sole function of improving quality. These include motor, neuro, and occupational rehabilitation which aim to improve muscular and neurological function in addition to improving overall cognition.

The purpose of this review article is to educate clinicians, healthcare workers, and most importantly the family of those who have been affected by this disease. It serves to shed light on this complex disorder and serve as a form of advocation for more research on identifying more etiologies, pathways of prevention, and reducing the burden of this disease for both patient and family.

## References

[1] Gulati S, Sondhi V (2018). Cerebral palsy: an overview. Indian J Pediatr.

[2] Vitrikas K, Dalton H, Breish D (2020). Cerebral palsy: an overview. Am Fam Physician.

[3] Smith KJ, Peterson MD, O'Connell NE (2020). Risk of depression and anxiety in adults with cerebral palsy. JAMA Neurol.

[4] Whitney DG, Warschausky SA, Peterson MD (2019). Mental health disorders and physical risk factors in children with cerebral palsy: a cross-sectional study. Dev Med Child Neurol.

[5] Kakooza A, Forssberg H, Eliasson A (2015). Cerebral palsy in children in Kampala, Uganda: clinical subtypes, motor function and co-morbidities. BMC Res Notes.

[6] MacLennan AH, Thompson SC, Gecz J (2015). Cerebral palsy: causes, pathways, and the role of genetic variants. Am J Obstet Gynecol.

[7] Wimalasundera N, Stevenson VL (2016). Cerebral palsy. Pract Neurol.

[8] Novak I, Morgan C, Adde L (2017). Early, accurate diagnosis and early intervention in cerebral palsy: advances in diagnosis and treatment. JAMA Pediatr.

[9] Marret S, Vanhulle C, Laquerriere A (2013). Pathophysiology of cerebral palsy. Handb Clin Neurol.

[10] Brandenburg JE, Fogarty MJ, Sieck GC (2019). A critical evaluation of current concepts in cerebral palsy. Physiology.

[11] Bearden D, Monokwane R, Khurana B (2016). Pediatric cerebral palsy in Botswana: etiology, outcomes, and comorbidities. Pediatr Neurol.

[12] Ellenberg JH, Nelson KB (2013). The association of cerebral palsy with birth asphyxia: a definitional quagmire. Dev Med Child Neurol.

[13] Duke R, Torty C, Nwachukwu K (2020). Clinical features and aetiology of cerebral palsy in children from Cross River State, Nigeria. Arch Dis Child.

[14] Reid SM, Carlin JB, Reddihough DS (2011). Distribution of motor types in cerebral palsy: how do registry data compare?. Dev Med Child Neurol.

[15] Gray L, Ng H, Bartlett D (2010). The gross motor function classification system: an update on impact and clinical utility. Pediatr Phys Ther.

[16] Paulson A, Vargus-Adams J (2017). Overview of four functional classification systems commonly used in cerebral palsy. Children.

[17] Ho PC, Chang CH, Granlund M (2017). The relationships between capacity and performance in youths with cerebral palsy differ for GMFCS levels. Pediatr Phys Ther.

[18] Minciu I (2012). Clinical correlations in cerebral palsy. Maedica.

[19] Imms C, Carlin J, Eliasson AC (2010). Stability of caregiver-reported manual ability and gross motor function classifications of cerebral palsy. Dev Med Child Neurol.

[20] Condliffe EG, Jeffery DT, Emery DJ (2016). Spinal inhibition and motor function in adults with spastic cerebral palsy. J Physiol.

[21] Sun D, Wang Q, Hou M (2018). Clinical characteristics and functional status of children with different subtypes of dyskinetic cerebral palsy. Medicine.

[22] Stanek JL, Emerson JA, Murdock FA (2016). Growth characteristics in cerebral palsy subtypes: a comparative assessment. Dev Med Child Neurol.

[23] Bell KL, Samson-Fang L (2013). Nutritional management of children with cerebral palsy. Eur J Clin Nutr.

[24] Herrera-Anaya E, Angarita-Fonseca A, Herrera-Galindo Herrera-Galindo (2016). Association between gross motor function and nutritional status in children with cerebral palsy: a cross-sectional study from Colombia. Dev Med Child Neurol.

[25] Kuperminc MN, Gottrand F, Samson-Fang L (2013). Nutritional management of children with cerebral palsy: a practical guide. Eur J Clin Nutr.

[26] Herskind A, Greisen G, Nielsen JB (2015). Early identification and intervention in cerebral palsy. Dev Med Child Neurol.

[27] Hadzagic-Catibusic F, Avdagic E, Zubcevic S (2017). Brain lesions in children with unilateral spastic cerebral palsy. Med Arch.

[28] Hadders-Algra M (2014). Early diagnosis and early intervention in cerebral palsy. Front Neurol.

[29] Romeo DM, Ricci D, Brogna C (2016). Use of the Hammersmith Infant Neurological Examination in infants with cerebral palsy: a critical review of the literature. Dev Med Child Neurol.

[30] Velde A, Morgan C, Novak I (2019). Early diagnosis and classification of cerebral palsy: an historical perspective and barriers to an early diagnosis. J Clin Med.

[31] Gibbins KJ, Browning KR, Lopes Lopes (2013). Evaluation of the clinical use of magnesium sulfate for cerebral palsy prevention. Obstet Gynecol.

[32] Basu AP (2014). Early intervention after perinatal stroke: opportunities and challenges. Dev Med Child Neurol.

[33] Shepherd E, Salam RA, Middleton P (2018). Neonatal interventions for preventing cerebral palsy: an overview of Cochrane Systematic Reviews. Cochrane Database Syst Rev.

[34] Soleimani F, Vameghi R, Biglarian A (2013). Antenatal and intrapartum risk factors for cerebral palsy in term and near-term newborns. Arch Iran Med.

[35] Panteliadis CP, Hagel C, Karch D (2015). Cerebral palsy: a lifelong challenge asks for early intervention. Open Neurol J.

[36] Sadowska M, Sarecka-Hujar B, Kopyta I (2020). Cerebral palsy: current opinions on definition, epidemiology, risk factors, classification and treatment options. Neuropsychiatr Dis Treat.

[37] Patel DR, Neelakantan M, Pandher K (2020). Cerebral palsy in children: a clinical overview. Transl Pediatr.

[38] Fairhurst C (2012). Cerebral palsy: the whys and hows. Arch Dis Child Educ Pract Ed.

[39] Byrne R, Noritz G, Maitre NL (2017). Implementation of early diagnosis and intervention guidelines for cerebral palsy in a high-risk infant follow-up clinic. Pediatr Neurol.

